# Biometric Identification of *Taxodium* spp. and Their Hybrid Progenies by Electrochemical Fingerprints

**DOI:** 10.3390/bios11100403

**Published:** 2021-10-18

**Authors:** Yuhong Zheng, Da Wang, Xiaolong Li, Ziyang Wang, Qingwei Zhou, Li Fu, Yunlong Yin, David Creech

**Affiliations:** 1 Jiangsu Engineering Research Center for Taxodium Rich, Germplasm Innovation and Propagation, Institute of Botany, Jiangsu Province and Chinese Academy of Sciences, Nanjing Botanical Garden, Memorial Sun Yat-Sen, Nanjing 210014, China; wangziyang@cnbg.net (Z.W.); ylyin@cnbg.net (Y.Y.); 2 College of Materials and Environmental Engineering, Hangzhou Dianzi University, Hangzhou 310018, China; wangda@hdu.edu.cn (D.W.); lxlr@hdu.edu.cn (X.L.); zhouqw@hdu.edu.cn (Q.Z.); 3 Arthur Temple College of Forestry and Agriculture, Stephen F. Austin State University, Nacogdoches, TX 75962, USA; dcreech@sfasu.edu

**Keywords:** electroanalysis, *Taxodium* spp., plant identification, fingerprints, biometrics

## Abstract

The use of electrochemical fingerprints for plant identification is an emerging application in biosensors. In this work, *Taxodium ascendens*, *T. distichum*, *T. mucronatum*, and 18 of their hybrid progenies were collected for this purpose. This is the first attempt to use electrochemical fingerprinting for the identification of plant hybrid progeny. Electrochemical fingerprinting in the leaves of *Taxodium* spp. was recorded under two conditions. The results showed that the electrochemical fingerprints of each species and progeny possessed very suitable reproducibility. These electrochemical fingerprints represent the electrochemical behavior of electrochemically active substances in leaf tissues under specific conditions. Since these species and progenies are very closely related to each other, it is challenging to identify them directly using a particular electrochemical fingerprinting. Therefore, electrochemical fingerprints measured under different conditions were used to perform pattern recognition. We can identify different species and progenies by locating the features in different pattern maps. We also performed a phylogenetic study with data from electrochemical fingerprinting. The results proved that the electrochemical classification results and the relationship between them are closely related.

## 1. Introduction

In order to improve the fast-growing traits and salinity tolerance of this genus for the development of forestry production and gardening, Chinese scientists have conducted interspecific hybridization tests since 1973. After more than two decades of efforts, the hybrid progeny named *T.* ‘Zhongshanshan’ was recognized by the State Forestry Administration in 2002. *T.* ‘Zhongshanshan’ has a very large number of cultivars, but the morphology is very similar among them. These different cultivars have trait differences between them and are suitable for different landscape applications [1,2]. For example, *T.* ‘Zhongshanshan (301)’ and *T.* ‘Zhongshanshan (302)’ are F1 generations that crossed between *T. distichum* (♀) and *T. mucronatum* (♂). *T.* ‘Zhongshanshan (401)’ is an F1 generation that crossed between *T. ascendens* (♀) and *T. mucronatum* (♂). Among them, *T.* ‘Zhongshanshan (302)’ has excellent ornamental value, high tolerance of water, moisture, and salinity. It can grow normally in soil pH below pH 8.5 and salinity below 0.3%. However, it is very difficult to identify different progenies by their morphology [3,4]. Therefore, it is necessary to develop a suitable biometric identification technique.

Electrochemical fingerprinting is a novel electroanalytical technique to obtain signals of electrochemically active components in plant tissues [5,6,7]. Electrochemical fingerprinting can show different curves due to the differences in the types and amounts of electrochemically active molecules contained in different plant tissues. These curves, although they can vary according to the environment and seasons, are mainly highly correlated with their species. Electrochemical fingerprinting techniques have been applied to different plant identifications in the last three years, but basically at genus level and species level. Its feasibility in identifying the progeny of different crosses is still worth exploring. In this work, we attempted to identify the 18 hybrid progenies of *T. distichum, T. ascendens, and T. mucronatum*. We found that although there were differences in electrochemical fingerprinting between the different hybrid progenies, such differences were significantly smaller than between different species. The identification of different hybrid progeny is difficult to reach from direct electrochemical fingerprints and requires pattern recognition of fingerprints under different conditions.

## 2. Materials and Methods

Leaves of *Taxodium ascendens*, *T. distichum*, *T. mucronatum*, and eighteen of their hybrid progenies were supplied by Nanjing Botanic Garden. All leaves were collected in April 2021. Table 1 shows the detailed information of all samples. When collecting, only mature and healthy leaves were harvested. All samples were kept frozen before analysis. Other reagents were analytical grade and used without further purification.

The extraction process was conducted using ethanol and water as solvents. Typically, 0.3 g leaves were chopped and added into 5 mL of solvent. Four grinding beads were added to the mixture. The tube was placed into a tissue grinding apparatus (Meibi-96, Zhejiang, China) for 2 min extraction. The extract was then collected through a 0.45 μm membrane.

The electrochemical recording process was according to a previous report [8]. The extraction process was conducted using ethanol or water as a solvent. A total of 0.1 M of phosphoric acid buffer solution (PBS, pH 7.0) and acetic acid buffer solution (ABS, pH 4.5) were used as supporting electrolytes. All electrochemical fingerprint recordings were conducted using a CHI760 electrochemical workstation (CH Instruments, Shanghai, China). A commercial glassy carbon electrode (GCE, 3 mm in diameter), an Ag/AgCl electrode, and a Pt electrode were used as the working electrode, reference electrode, and counter electrode, respectively. Each sample was collected three times in parallel. A differential pulse voltammetry (DPV) was applied as a scan method in recording the electrochemical fingerprint. The scan range is between 0 and 1.3 V (pulse amplitude: 50 mV; pulse width: 0.05 s; pulse period: 0.5 s).

2D density map and heatmap were generated using Origin based on the data of electrochemical fingerprint. A heat map is a data visualization technique that shows the magnitude of a phenomenon as color in two dimensions. The 2D density map is a smoothed color density representation of the scatterplot, based on kernel density estimation, a nonparametric technique for probability density functions. Density values are calculated based on the equation below:

(1) $$f\left(x,y,\mathrm{vX},\mathrm{vY},\omega_{x},\omega_{y}\right)=\frac{1}{n}\overset{n}{\underset{i=1}{\sum }}\frac{1}{2{\pi \omega }_{x}\omega_{y}}\exp(-\frac{{\left(x-{\mathrm{vX}}_{i}\right)}^{2}}{2\omega_{x}^{2}}-\frac{{\left(y-{\mathrm{vY}}_{i}\right)}^{2}}{2\omega_{y}^{2}})$$

A normalization process was conducted for all recorded electrochemical fingerprints [9], where the ratios between the current and the maximum peak current were obtained at different potentials.

## 3. Results and Discussion

**Figure 1:**
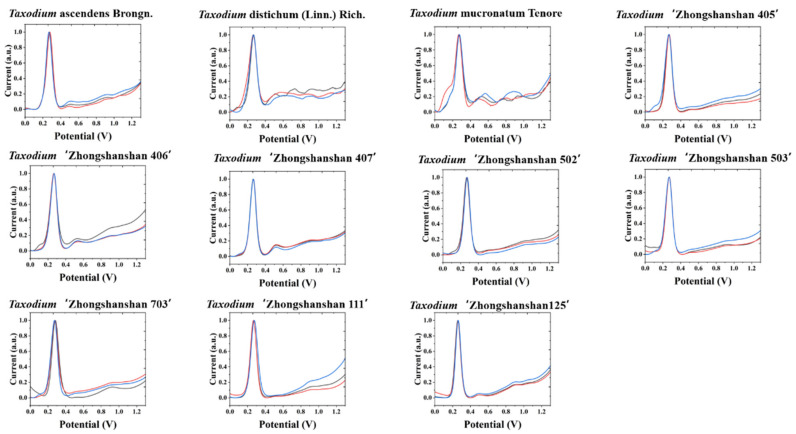
Electrochemical fingerprint of *T. ascendens*, *T. distichum*, *T. mucronatum*, *T.* ‘Zhongshanshan 405’, *T.* ‘Zhongshanshan 406’, *T.* ‘Zhongshanshan 407’, *T.* ‘Zhongshanshan 502’, *T.* ‘Zhongshanshan 503’, *T.* ‘Zhongshanshan 703’, *T.* ‘Zhongshanshan 111’, and *T.* ‘Zhongshanshan 125’ after water extraction and recorded under PBS condition.

**Figure 2:**
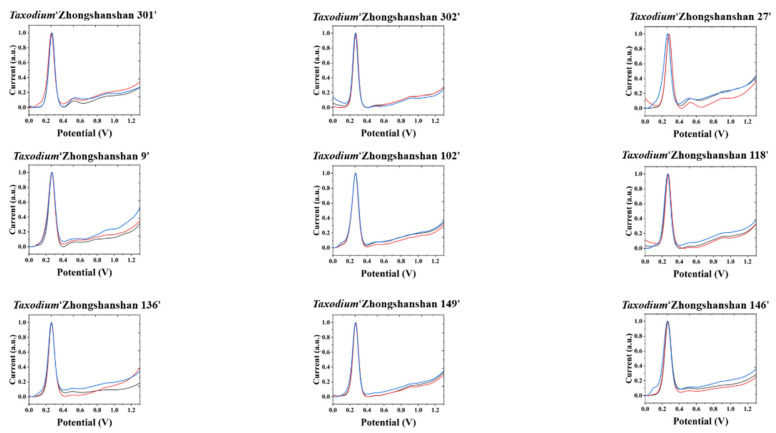
Electrochemical fingerprint of *T.* ‘Zhongshanshan 301’, *T.* ‘Zhongshanshan 302’, *T.* ‘Zhongshanshan 27’, *T.* ‘Zhongshanshan 9’, *T.* ‘Zhongshanshan 102’, *T.* ‘Zhongshanshan 118’, *T.* ‘Zhongshanshan 136’, *T.* ‘Zhongshanshan 125’, and *T.* ‘Zhongshanshan 146’ after water extraction and recorded under PBS condition.

The electrochemical fingerprints of all samples after water extraction recorded under PBS conditions are shown in Figure 1 and Figure 2. In the scanning window, a series of oxidation peaks can be observed. These oxidation peaks represent oxidation reactions of electrochemically active molecules in plant leaf tissues during voltammetric scanning. According to previous studies, these molecules are mainly flavanols [10], phenolic acids [11], procyanidins [12], alkaloids [13], and pigments [14]. According to our previous studies on electroanalytical chemistry and phytochemistry [15,16,17,18], the substances that undergo electrochemical oxidation at 0.3 V are most likely ascorbic acid and luteolin. There are many possibilities for other oxidation peaks between 0.4 and 1 V. In our experience, substances that can be identified include catechin and coumarin [19]. Based on reports from others, these peaks also contain quercetin, morin, and cadinene [20,21,22]. The differences in peak potentials are often due to differences in the structure of the molecules involved in the reaction [23]. From the voltammetric curve, all three species of *Taxodium* have a very obvious oxidation peak. In addition, there are some relatively small oxidation peaks above 0.4 V. We conducted MANOVA tests for our data. The *p*–values of the variables recorded for three individual electrochemical fingerprints from the same species or progeny all larger than 0.05, indicating no significant differences within the species or progeny. However, when comparing different species and progeny, the *p*-value of 3.4 × 10^−7^, suggesting a significant difference. Therefore, the differences in electrochemical fingerprints between the species and progeny are much larger than the natural variability of one species. Compared to some previous literature [24,25,26,27], the electrochemical fingerprints of Taxodium species were not very different from each other. Different scientists have different views on the genetics of *Taxodium*. Some scientists consider *T. ascendens* to be a different species compared to *T. distichum*, while others consider *T. ascendens* to be a species or ecotype of *T. distichum*. Therefore, the similarity of electrochemical fingerprinting also represents that they are relatively similar at the genetic level. At the same time, we can see that the different cultivars of *T. ‘Zhongshanshan’* are very similar to each other. Based on the above observations, it is difficult to identify different species directly with a single electrochemical fingerprint. Therefore, the identification of different cultivars of *T. ‘Zhongshanshan’* is a more difficult challenge.

If electrochemical fingerprinting is recorded for each sample under different conditions, the abundance of the signal can be increased. Different electrochemically active molecules in plant tissues show different electrochemical behavior under different pH and electrolytes. Therefore, we collected fingerprints for each sample in ABS as well (Figures S1 and S2). In addition, the solvent used for the extraction is also important because the molecules involved in the electrochemical reaction depend on the outcome of the extraction. Therefore, this work also used ethanol as a solvent for the extraction of plant tissues. The ethanol extracts of plants were also recorded in PBS (Figures S3 and S4) and ABS (Figures S5 and S6) for electrochemical fingerprinting. It can be seen that the electrochemical behavior of plant tissues, whether they are extracted with water or ethanol, will be more different at ABS. This represents a richer electrochemical oxidation behavior of the electrochemically active molecules in these samples in a low pH environment [28]. The most important reason why more signals are collected in ABS than in PBS is that more electrochemically active substances will be involved in the reaction during the scan interval. This is due to the fact that the electrochemical behavior with the involvement of protons receives the influence of pH conditions. A decrease in pH shifts the peak position of electrochemical oxidation negatively. Therefore, in our scan interval, ABS (pH 4.5) conditions will have more electrochemically active substances involved in the oxidation than PBS (pH 7.0). However, the oxidation current of the same electrochemically active substance does not necessarily have a very direct relationship with pH change. Usually, electrochemically active substances will only show a small signal in a too acidic environment; therefore, we chose ABS (pH 4.5) as the recording condition. These differences in electrochemical oxidation behavior reflect the degree of variability of the electrochemically active substances involved. At the same time, we perceived that the electrochemical fingerprints collected in ABS would have a large variation. Although the positions of the oxidation peaks are almost identical, the peak currents can be different. This represents that the amount of electrochemically active molecules involved in these oxidation behaviors may not be the same for every sample, even in the same species or variety. These behavioral differences may be due to cumulative differences in composition caused by different growth environments [29].

**Figure 3:**
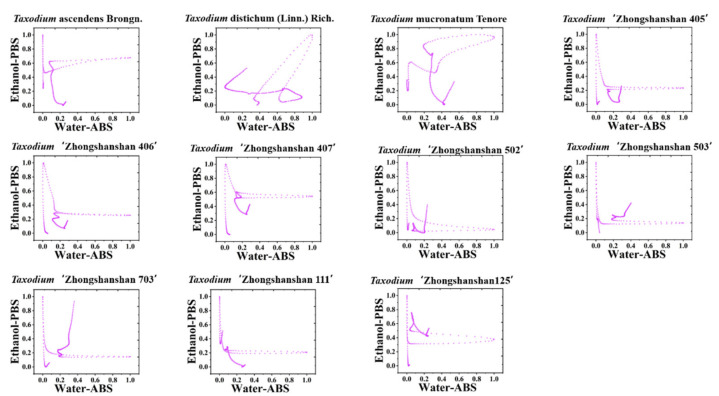
Scatter plots of *T.* ascendens, *T.* distichum, *T.* mucronatum, *T.* ‘Zhongshanshan 405’, *T.* ‘Zhongshanshan 406’, *T.* ‘Zhongshanshan 407’, *T.* ‘Zhongshanshan 502’, *T.* ‘Zhongshanshan 503’, *T.* ‘Zhongshanshan 703’, *T.* ‘Zhongshanshan 111’, and *T.* ‘Zhongshanshan 125’ combining the signals collected under ABS for the water extracts and under PBS for the ethanol extracts.

Since the *X*-axis of electrochemical fingerprinting is potential, it does not contribute weight in sample-to-sample comparisons. Therefore, we can combine the electrochemical fingerprints from two different conditions to form a suitable pattern. For example, Figure 3 and Figure S7 show scatter plots combining the signals collected under ABS for the water extracts and under PBS for the ethanol extracts. It can be seen that the accuracy of identifying them can be increased. The originally similar electrochemical fingerprint profiles can be easily identified in the scatter plot. The abundance of the signal increases with increasing values on the x- and y-axes. The farther the point in the scatter plot is from the origin means that it has a stronger signal in the original electrochemical fingerprint profile. If the data point is near the upper right corner of the scatter plot, it means that the plant extract has a very strong signal at the potential of that point, regardless of the conditions under which the electrochemical fingerprint was collected. Therefore, data points far from the origin in the scatter plot can be used as characteristic markers of the sample for identification. For example, the electrochemical fingerprints of water extracts of *T. ‘Zhongshanshan 111’ and T. ‘Zhongshanshan 125’* were very similar under ABS, but significant differences could be detected in the scatter plot. In addition, electrochemical fingerprints of water extracts of *T. ‘Zhongshanshan 302’, T. ‘Zhongshanshan 102’*, and *T. ‘Zhongshanshan 149’* were very similar under ABS but also showed significant differences in the scatter plots.

**Figure 4:**
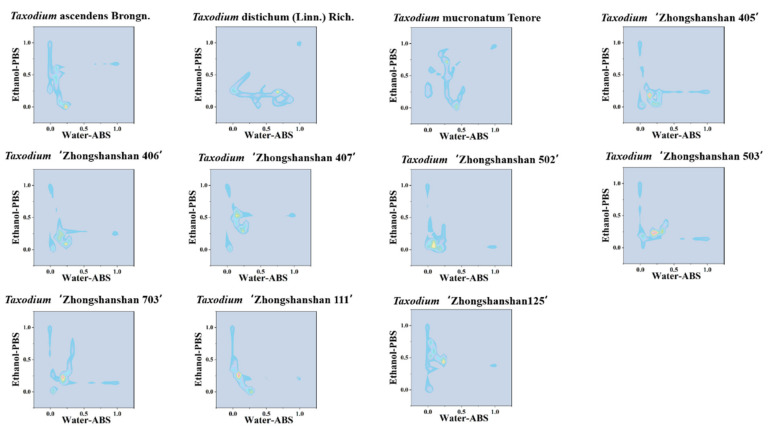
Two-dimensional density map of *T.* ascendens, *T.* distichum, *T.* mucronatum, *T.* ‘Zhongshanshan 405’, *T.* ‘Zhongshanshan 406’, *T.* ‘Zhongshanshan 407’, *T.* ‘Zhongshanshan 502’, *T.* ‘Zhongshanshan 503’, *T.* ‘Zhongshanshan 703’, *T.* ‘Zhongshanshan 111’, and *T.* ‘Zhongshanshan 125’ combining the signals collected under ABS for the water extracts and under PBS for the ethanol extracts.

**Figure 5:**
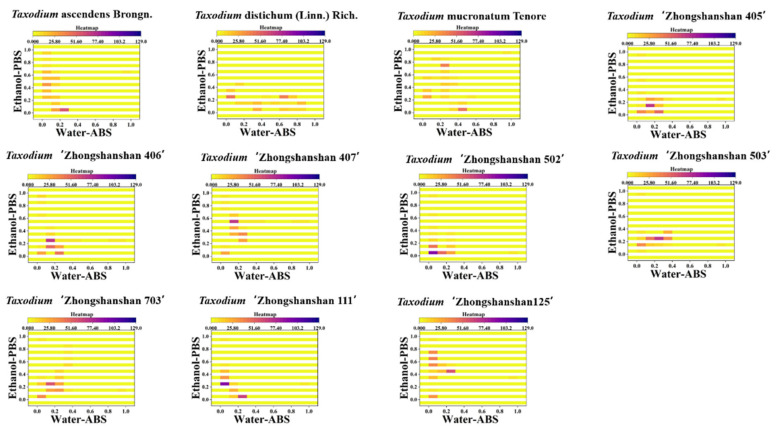
Heatmap of *T.* ascendens, *T.* distichum, *T.* mucronatum, *T.* ‘Zhongshanshan 405’, *T.* ‘Zhongshanshan 406’, *T.* ‘Zhongshanshan 407’, *T.* ‘Zhongshanshan 502’, *T.* ‘Zhongshanshan 503’, *T*. ‘Zhongshanshan 703’, *T.* ‘Zhongshanshan 111’, and *T.* ‘Zhongshanshan 125’ combining the signals collected under ABS for the water extracts and under PBS for the ethanol extracts.

Although the scatter plot can represent the difference between different samples more intuitively than a single electrochemical fingerprint, it is not very easy to count the scatter points in the plot directly. Therefore, we can use a two-dimensional density map to strengthen the weights of some data points. In a two-dimensional density map (Figure 4 and Figure S8), data points that are clustered closer together will appear in a darker color. Therefore, we can achieve the identification of species based on locating the position of these key regions [30]. The advantage of this pattern recognition is that the amount of data for image recognition can be reduced. In addition, the heat map also can be used to obviously calculate the similarity between different samples (Figure 5 and Figure S9). Although these pattern recognition approaches are all based on data from electrochemical fingerprinting, they use different recognition strategies. The heat map combines the advantages of scatter plot and 2D density map, which not only locates the density of the data but also segments the data points by a reasonable grid, which is more suitable for accurate identification. These patterns generated based on electrochemical fingerprinting can be used for plant identification because electrochemical fingerprinting contains information about electrochemically active molecules in plant tissues [31]. This information mainly responds to the synthesis and accumulation of phytochemicals due to the difference at the genetic level.

**Figure 6:**
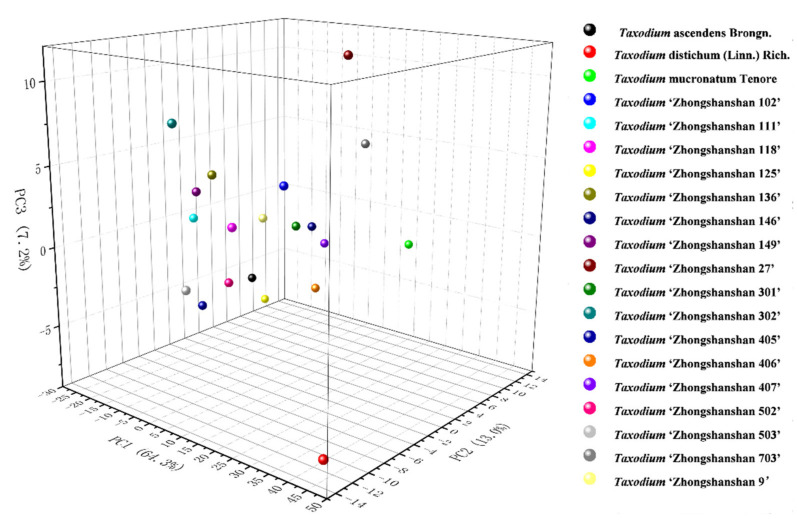
PCA analysis of *T. ascendens*, *T. distichum*, *T. mucronatum*, *T.* ‘Zhongshanshan 405’, *T.* ‘Zhongshanshan 406’, *T.* ‘Zhongshanshan 407’, *T.* ‘Zhongshanshan 502’, *T.* ‘Zhongshanshan 503’, *T.* ‘Zhongshanshan 703’, *T.* ‘Zhongshanshan 111’, *T.* ‘Zhongshanshan 125’, *T.* ‘Zhongshanshan 301’, *T.* ‘Zhongshanshan 302’, *T.* ‘Zhongshanshan 27’, *T.* ‘Zhongshanshan 9’, *T.* ‘Zhongshanshan 102’, *T.* ‘Zhongshanshan 118’, *T.* ‘Zhongshanshan 136’, *T.* ‘Zhongshanshan 125’, and *T.* ‘Zhongshanshan 146’.

Since the information from electrochemical fingerprinting can respond to gene-level differences, we tried to statistically analyze all the samples using principal component analysis (PCA). Since this study contains 20 groups and we chose 3D PCA (3 components), this makes the points in the final graph very crowded and makes the relationships between them very ambiguous. Therefore, for a clearer reading experience, we have chosen a point corresponding to a species or hybrid progeny. Values 108.27598, 9.32409, and 1.76683 were three extracted eigenvalues. As shown in Figure 6, after extracting three factors, PCA could reach 84.5% interpretation. Thus, the electrochemical fingerprint contains representative information points that can be used to represent different data sets. This further indicated that electrochemical fingerprinting can be used for the identification of species of *Taxodium* and their hybrid progenies.

**Figure 7:**
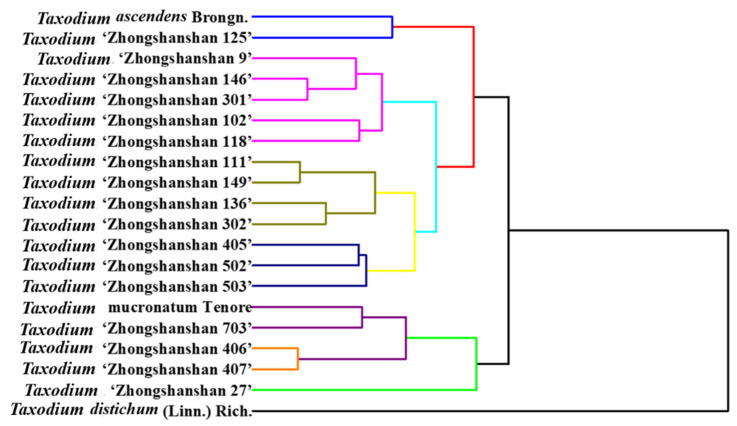
Dendrogram of *T. ascendens*, *T. distichum*, *T. mucronatum*, *T.* ‘Zhongshanshan 405’, *T.* ‘Zhongshanshan 406’, *T.* ‘Zhongshanshan 407’, *T.* ‘Zhongshanshan 502’, *T.* ‘Zhongshanshan 503’, *T.* ‘Zhongshanshan 703’, *T.* ‘Zhongshanshan 111’, *T.* ‘Zhongshanshan 125’, *T.* ‘Zhongshanshan 301’, *T.* ‘Zhongshanshan 302’, *T.* ‘Zhongshanshan 27’, *T.* ‘Zhongshanshan 9’, *T.* ‘Zhongshanshan 102’, *T.* ‘Zhongshanshan 118’, *T.* ‘Zhongshanshan 136’, *T.* ‘Zhongshanshan 125’, and *T.* ‘Zhongshanshan 146’ based on electrochemical fingerprints.

Due to the high frequency of using a few backbone parents in the breeding process, some of the *T. ‘Zhongshanshan’* cultivars are closely related. The differences in morphological characters are small, making it difficult to identify them. The cultivars of *T. ‘Zhongshanshan’* have been investigated by several works using morphological and molecular characteristics [32,33,34]. The results showed that by using the differences in external morphological traits, the clustering analysis could only sort out the affinities among some species, cultivars, and hybrids. The phenotypic characters were easily influenced by environmental factors [35]. The use of RAPD molecules for relationship study is another strategy. However, RAPD molecular markers provide dominant markers and cannot distinguish between pure and heterozygous types. Therefore, the genetic information obtained by this method is not comprehensive [36]. Here, we performed a clustering analysis among different species and cultivars using electrochemical fingerprinting. As shown in Figure 7, the entire phylogenetic tree is divided into five main clades. The first clade contains *T. ascendens* and *T.* ‘Zhongshanshan 125’. The second clade contains *T.* ‘Zhongshanshan 9’, *T.* ‘Zhongshanshan 146’, *T.* ‘Zhongshanshan 301’, *T.* ‘Zhongshanshan 102’ and *T.* ‘Zhongshanshan 118’. The third clade contains *T.* ‘Zhongshanshan 111’, *T.* ‘Zhongshanshan 149’, *T.* ‘Zhongshanshan 136’ and *T.* ‘Zhongshanshan 302’. The fourth clade contains *T.* ‘Zhongshanshan 405’, *T.* ‘Zhongshanshan 502’, and *T.* ‘Zhongshanshan 503’. The last clade contains *T. mucronatum, T.* ‘Zhongshanshan 703’, *T.* ‘Zhongshanshan 406’, and *T.* ‘Zhongshanshan 407’. In addition, *T.* ‘Zhongshanshan 27’ and *T. distichum* are not in these main clades. A very suitable agreement between the results of the cluster analysis and the actual relatives can be observed by comparing Table 1. For example, *T.* ‘Zhongshanshan 405’, *T.* ‘Zhongshanshan 406’, *T.* ‘Zhongshanshan 407‘, *T.* ‘Zhongshanshan 502’, *T.* ‘Zhongshanshan 503’ and *T*. ‘Zhongshanshan 703’ all clustered in very close proximity to each other. Their parents are both *T. mucronatum* and *T. distichum*, and they are all progenies of the reciprocal cross. In addition, *T. ‘Zhongshanshan 9’, T.* ‘Zhongshanshan 27’, *T*. ‘Zhongshanshan 102‘, *T.* ‘Zhongshanshan 118’, *T.* ‘Zhongshanshan 136’, *T.* ‘Zhongshanshan 146’, and *T.* ‘Zhongshanshan 149’ all clustered in very close proximity to each other. Their parents are both *T.* ‘Zhongshanshan 302’ and *T. mucronatum*, and they are all progenies of backcross. We also perceive some results from the graph that are different from Table 1. For example, *T.* ‘Zhongshanshan 111’ and *T.* ‘Zhongshanshan 125’ are in relatively distant positions, but they are both cross progenies of *T. mucronatum* and *T. ascendens*. These different results may be due to the fact that our electrochemical acquisition signal is not comprehensive enough. We chose only two buffer environments, acidic and neutral, and could not obtain a full profile of electrochemically active substances in plant tissue. In the meantime, we used only two extraction solvents, which did not completely extract the electrochemically active molecules from the plant tissues. Therefore, these electrochemical fingerprints remain a little defective in terms of abundance. These shortcomings will be optimized in our future work.

## 4. Conclusions

In conclusion, the electrochemical fingerprint of the *T. ascendens*, *T. distichum*, *T. mucronatum*, and 18 of their hybrid progenies were recorded using water and ethanol extracts under PBS and ABS. These electrochemical fingerprints can be used for species and cultivars identification. Pattern recognition approaches constructed using these fingerprints can be used more effectively for different identification strategies. The electrochemical fingerprint signal can also be used as a signal ensemble to study the relationship between different species and cultivars.

## Figures and Tables

**Table 1:** Sample information of *T. ascendens*, *T. distichum*, *T. mucronatum*, and eighteen of their hybrid progenies used in this work.

No.	Cultivars	Female Parent	Male Parent	Note
1	*Taxodium* ‘Zhongshanshan 301’	*T. distichum*	*T. mucronatum*	Cross
2	*Taxodium* ‘Zhongshanshan 302’	*T. distichum*	*T. mucronatum*	Cross
3	*Taxodium* ‘Zhongshanshan 405’	*T. mucronatum*	*T. distichum*	Reciprocal cross
4	*Taxodium* ‘Zhongshanshan 406’	*T. mucronatum*	*T. distichum*	Reciprocal cross
5	*Taxodium* ‘Zhongshanshan 407’	*T. mucronatum*	*T. distichum*	Reciprocal cross
6	*Taxodium* ‘Zhongshanshan 502’	*T. mucronatum*	*T. distichum*	Reciprocal cross
7	*Taxodium* ‘Zhongshanshan 503’	*T. mucronatum*	*T. distichum*	Reciprocal cross
8	*Taxodium* ‘Zhongshanshan 703’	*T. mucronatum*	*T. distichum*	Reciprocal cross
9	*Taxodium* ‘Zhongshanshan 9’	*Taxodium* ‘Zhongshanshan 302’	*T. mucronatum*	Backcross
10	*Taxodium* ‘Zhongshanshan 27’	*Taxodium* ‘Zhongshanshan 302’	*T. mucronatum*	Backcross
11	*Taxodium* ‘Zhongshanshan 102’	*Taxodium* ‘Zhongshanshan 302’	*T. mucronatum*	Backcross
12	*Taxodium* ‘Zhongshanshan 118’	*Taxodium* ‘Zhongshanshan 302’	*T. mucronatum*	Backcross
13	*Taxodium* ‘Zhongshanshan 136’	*Taxodium* ‘Zhongshanshan 302’	*T. mucronatum*	Backcross
14	*Taxodium* ‘Zhongshanshan 146’	*Taxodium* ‘Zhongshanshan 302’	*T. mucronatum*	Backcross
15	*Taxodium* ‘Zhongshanshan 149’	*Taxodium* ‘Zhongshanshan 302’	*T. mucronatum*	Backcross
16	*Taxodium* ‘Zhongshanshan 111’	*T. mucronatum*	*T. ascendens*	Cross
17	*Taxodium* ‘Zhongshanshan 125’	*T. mucronatum*	*T. ascendens*	Cross
18	*Taxodium* ‘Zhongshanshan 401’	*T. ascendens*	*T. mucronatum*	Reciprocal cross

## Data Availability

Not applicable.

## Supplementary Materials

The following are available online at https://www.mdpi.com/article/10.3390/bios11100403/s1, Figure S1: Electrochemical fingerprint of *T. ascendens*, *T. distichum*, *T. mucronatum*, *T.* ‘Zhongshanshan 405’, *T.* ‘Zhongshanshan 406’, *T.* ‘Zhongshanshan 407’, *T.* ‘Zhongshanshan 502’, *T.* ‘Zhongshanshan 503’, *T.* ‘Zhongshanshan 703’, *T.* ‘Zhongshanshan 111’ and and *T.* ‘Zhongshanshan 125’ after water extraction and recorded under ABS condition. Figure S2: Electrochemical fingerprint of *T.* ‘Zhongshanshan 301’, *T.* ‘Zhongshanshan 302’, *T.* ‘Zhongshanshan 27’, *T.* ‘Zhongshanshan 9’, *T.* ‘Zhongshanshan 102’, *T.* ‘Zhongshanshan 118’, *T.* ‘Zhongshanshan 136’, *T.* ‘Zhongshanshan 125’ and *T.* ‘Zhongshanshan 146’ after water extraction and recorded under ABS condition. Figure S3: Electrochemical fingerprint of *T.* ascendens, *T.* distichum, *T.* mucronatum, *T.* ‘Zhongshanshan 405’, *T.* ‘Zhongshanshan 406’, *T.* ‘Zhongshanshan 407’, *T.* ‘Zhongshanshan 502’, *T.* ‘Zhongshanshan 503’, *T.* ‘Zhongshanshan 703’, *T.* ‘Zhongshanshan 111’ and *T.* ‘Zhongshanshan 125’ after water extraction and recorded under PBS condition. Figure S4: Electrochemical fingerprint of *T.* ‘Zhongshanshan 301’, *T.* ‘Zhongshanshan 302’, *T.* ‘Zhongshanshan 27’, *T.* ‘Zhongshanshan 9’, *T.* ‘Zhongshanshan 102’, *T.* ‘Zhongshanshan 118’, *T.* ‘Zhongshanshan 136’, *T.* ‘Zhongshanshan 125’ and *T.* ‘Zhongshanshan 146’ after water water and recorded under ABS condition. Figure S5: Electrochemical fingerprint of *T.* ascendens, *T.* distichum, *T.* mucronatum, *T.* ‘Zhongshanshan 405’, *T.* ‘Zhongshanshan 406’, *T.* ‘Zhongshanshan 407’, *T.* ‘Zhongshanshan 502’, *T.* ‘Zhongshanshan 503’, *T.* ‘Zhongshanshan 703’, *T.* ‘Zhongshanshan 111’ and *T.* ‘Zhongshanshan 125’ after ethanol extraction and recorded under ABS condition. Figure S6: Electrochemical fingerprint of *T.* ‘Zhongshanshan 301’, *T.* ‘Zhongshanshan 302’, *T.* ‘Zhongshanshan 27’, *T.* ‘Zhongshanshan 9’, *T.* ‘Zhongshanshan 102’, *T.* ‘Zhongshanshan 118’, *T.* ‘Zhongshanshan 136’, *T.* ‘Zhongshanshan 125’ and *T.* ‘Zhongshanshan 146’ after water ethanol and recorded under ABS condition. Figure S7: Scatter plots of *T.* ‘Zhongshanshan 301’, *T.* ‘Zhongshanshan 302’, *T.* ‘Zhongshanshan 27’, *T.* ‘Zhongshanshan 9’, *T.* ‘Zhongshanshan 102’, *T.* ‘Zhongshanshan 118’, *T.* ‘Zhongshanshan 136’, *T.* ‘Zhongshanshan 125’ and *T.* ‘Zhongshanshan 146’ combining the signals collected under ABS for the water extracts and under PBS for the ethanol extracts. Figure S8: 2D density map of *T.* ‘Zhongshanshan 301’, *T.* ‘Zhongshanshan 302’, *T.* ‘Zhongshanshan 27’, *T.* ‘Zhongshanshan 9’, *T.* ‘Zhongshanshan 102’, *T.* ‘Zhongshanshan 118’, *T.* ‘Zhongshanshan 136’, *T.* ‘Zhongshanshan 125’ and *T.* ‘Zhongshanshan 146’ combining the signals collected under ABS for the water extracts and under PBS for the ethanol extracts. Figure S9: Heatmap of *T.* ‘Zhongshanshan 301’, *T.* ‘Zhongshanshan 302’, *T.* ‘Zhongshanshan 27’, *T.* ‘Zhongshanshan 9’, *T.* ‘Zhongshanshan 102’, *T.* ‘Zhongshanshan 118’, *T.* ‘Zhongshanshan 136’, *T.* ‘Zhongshanshan 125’ and *T.* ‘Zhongshanshan 146’ combining the signals collected under ABS for the water extracts and under PBS for the ethanol extracts.
