# Supplementary material for: Biometric Identification of Taxodium spp. and Their Hybrid Progenies by Electrochemical Fingerprints
Source: Biosensors (Basel). 2021 Oct 18;11(10):403. doi: 10.3390/bios11100403 (PMC8534068; doi:10.3390/bios11100403)
Supplement: Supplementary file 1 [file biosensors-11-00403-s001.zip › biosensors-1395816-supplementary.pdf]

biosensors

Supplementary Materials

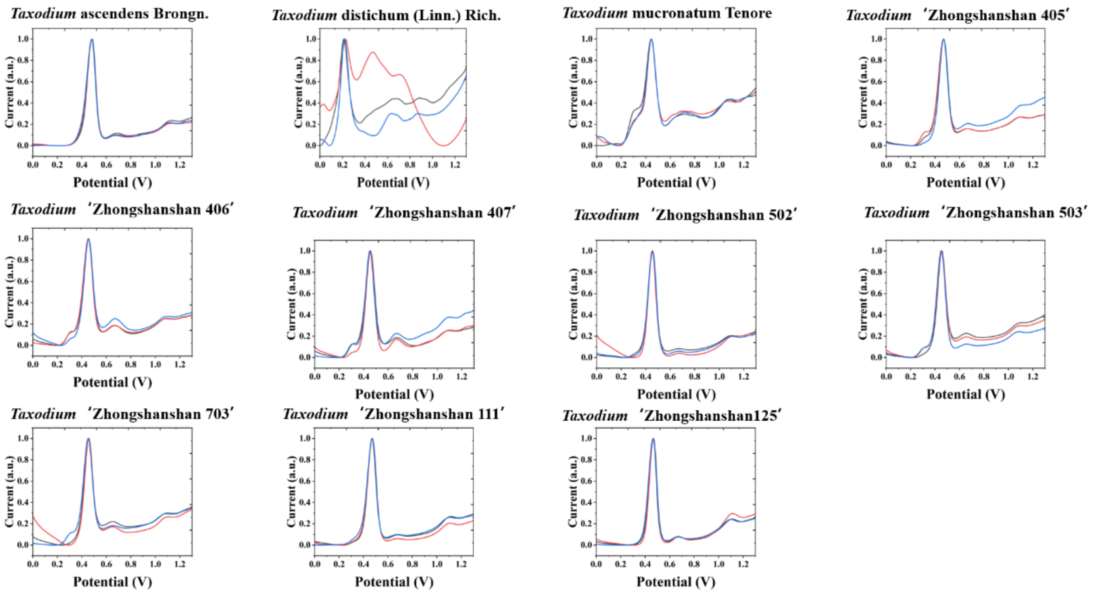

Figure S1. Electrochemical fingerprint of *T.* ascendens, *T.* distichum, *T.* mucronatum, *T.* 'Zhongshanshan 405', *T.* 'Zhongshanshan 406', *T.* 'Zhongshanshan 407', *T.* 'Zhongshanshan 502', *T.* 'Zhongshanshan 503', *T.* 'Zhongshanshan 703', *T.* 'Zhongshanshan 111' and *T.* 'Zhongshanshan 125' after water extraction and recorded under ABS condition.

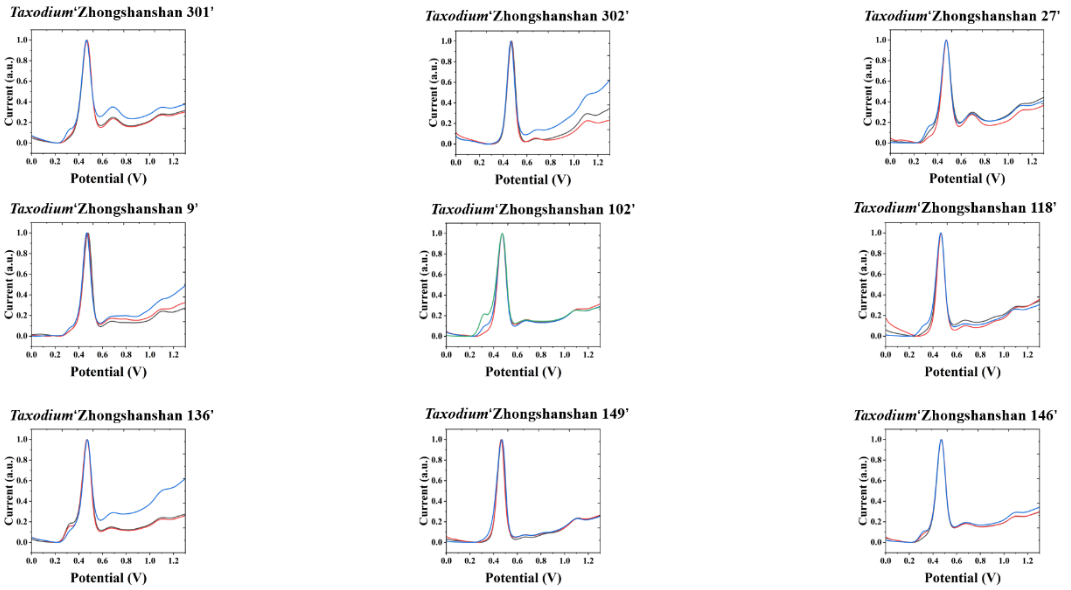

Figure S2. Electrochemical fingerprint of *T.* 'Zhongshanshan 301', *T.* 'Zhongshanshan 302', *T.* 'Zhongshanshan 27', *T.* 'Zhongshanshan 9', *T.* 'Zhongshanshan 102', *T.* 'Zhongshanshan 118', *T.* 'Zhongshanshan 136', *T.* 'Zhongshanshan 125' and *T.* 'Zhongshanshan 146' after water extraction and recorded under ABS condition.

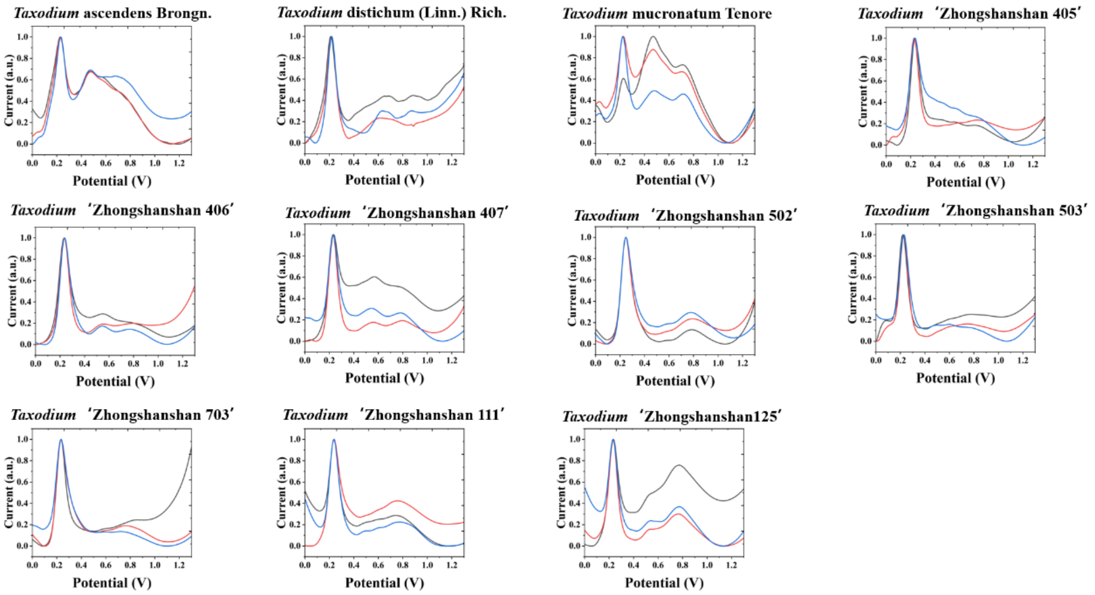

Figure S3. Electrochemical fingerprint of *T*. ascendens, *T*. distichum, *T*. mucronatum, *T*. 'Zhongshanshan 405', *T*. 'Zhongshanshan 406', *T*. 'Zhongshanshan 407', *T*. 'Zhongshanshan 502', *T*. 'Zhongshanshan 503', *T*. 'Zhongshanshan 703', *T*. 'Zhongshanshan 111' and *T*. 'Zhongshanshan 125' after water extraction and recorded under PBS condition.

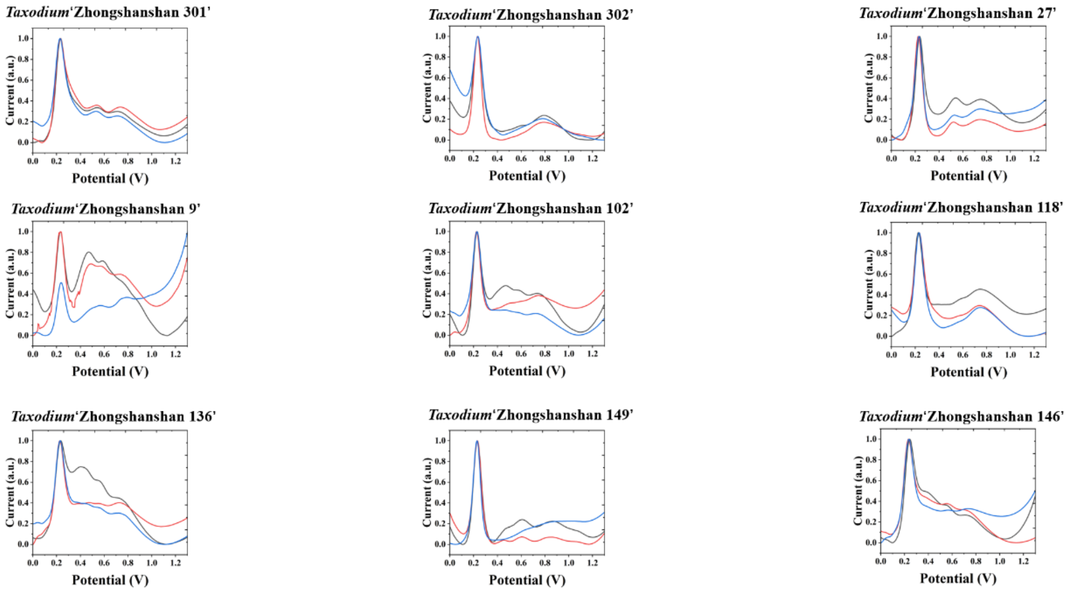

Figure S4. Electrochemical fingerprint of *T*. 'Zhongshanshan 301', *T*. 'Zhongshanshan 302', *T*. 'Zhongshanshan 27', *T*. 'Zhongshanshan 9', *T*. 'Zhongshanshan 102', *T*. 'Zhongshanshan 118', *T*. 'Zhongshanshan 136', *T*. 'Zhongshanshan 125' and *T*. 'Zhongshanshan 146' after water water and recorded under ABS condition.

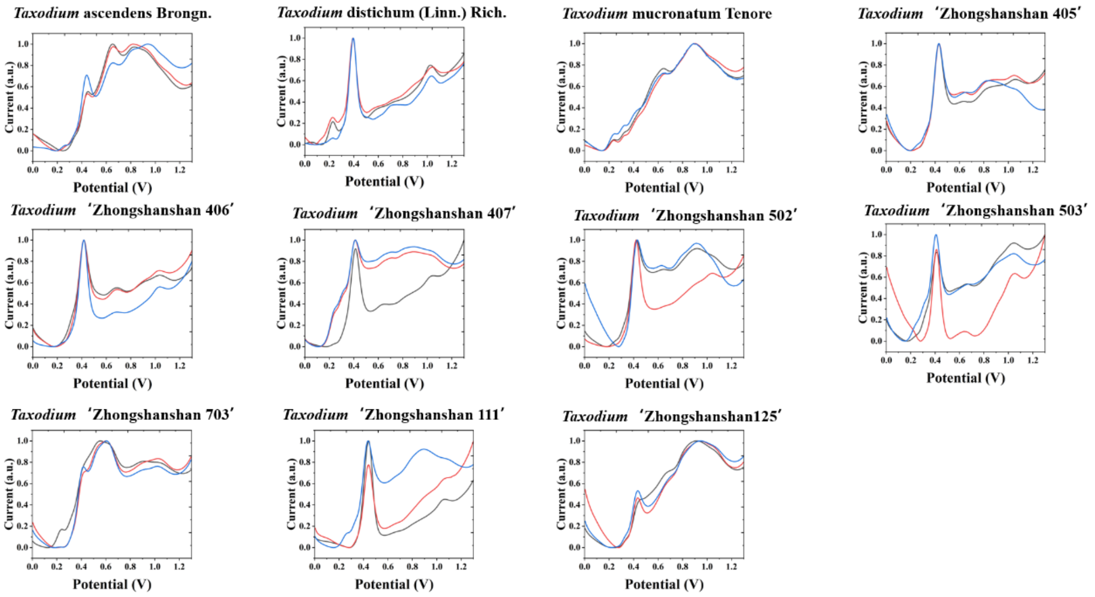

Figure S5. Electrochemical fingerprint of *T*. ascendens, *T*. distichum, *T*. mucronatum, *T*. 'Zhongshanshan 405′, *T*. 'Zhongshanshan 406′, *T*. 'Zhongshanshan 407′, *T*. 'Zhongshanshan 502′, *T*. 'Zhongshanshan 503′, *T*. 'Zhongshanshan 703′, *T*. 'Zhongshanshan 111′ and *T*. 'Zhongshanshan 125′ after ethanol extraction and recorded under ABS condition.

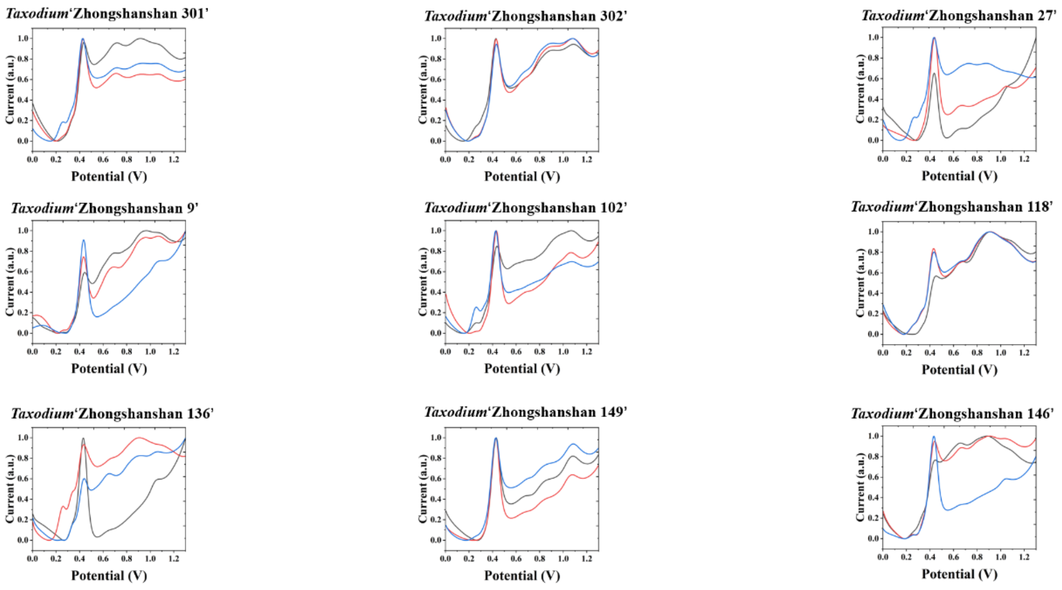

Figure S6. Electrochemical fingerprint of *T*. 'Zhongshanshan 301′, *T*. 'Zhongshanshan 302′, *T*. 'Zhongshanshan 27′, *T*. 'Zhongshanshan 9′, *T*. 'Zhongshanshan 102′, *T*. 'Zhongshanshan 118′, *T*. 'Zhongshanshan 136′, *T*. 'Zhongshanshan 125′ and *T*. 'Zhongshanshan 146′ after water ethanol and recorded under ABS condition.

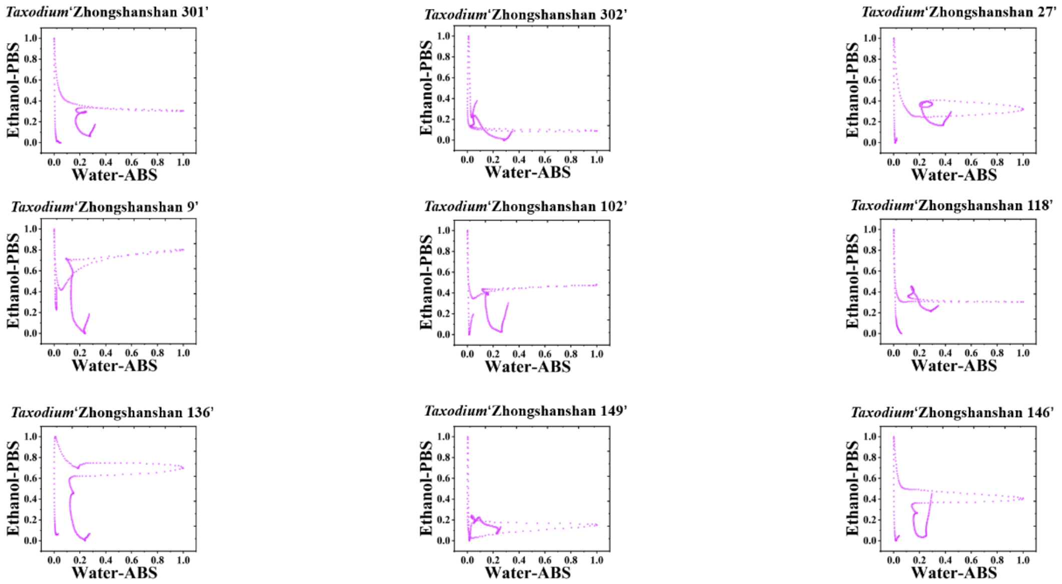

Figure S7. Scatter plots of *T*. 'Zhongshanshan 301', *T*. 'Zhongshanshan 302', *T*. 'Zhongshanshan 27', *T*. 'Zhongshanshan 9', *T*. 'Zhongshanshan 102', *T*. 'Zhongshanshan 118', *T*. 'Zhongshanshan 136', *T*. 'Zhongshanshan 125' and *T*. 'Zhongshanshan 146' combining the signals collected under ABS for the water extracts and under PBS for the ethanol extracts.

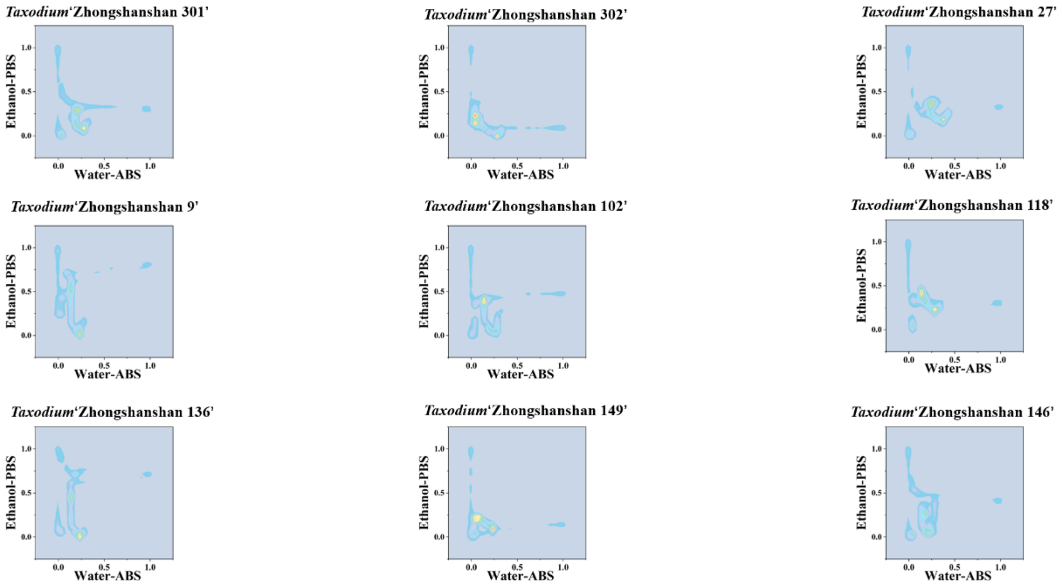

Figure S8. 2D density map of *T*. 'Zhongshanshan 301', *T*. 'Zhongshanshan 302', *T*. 'Zhongshanshan 27', *T*. 'Zhongshanshan 9', *T*. 'Zhongshanshan 102', *T*. 'Zhongshanshan 118', *T*. 'Zhongshanshan 136', *T*. 'Zhongshanshan 125' and *T*. 'Zhongshanshan 146' combining the signals collected under ABS for the water extracts and under PBS for the ethanol extracts.

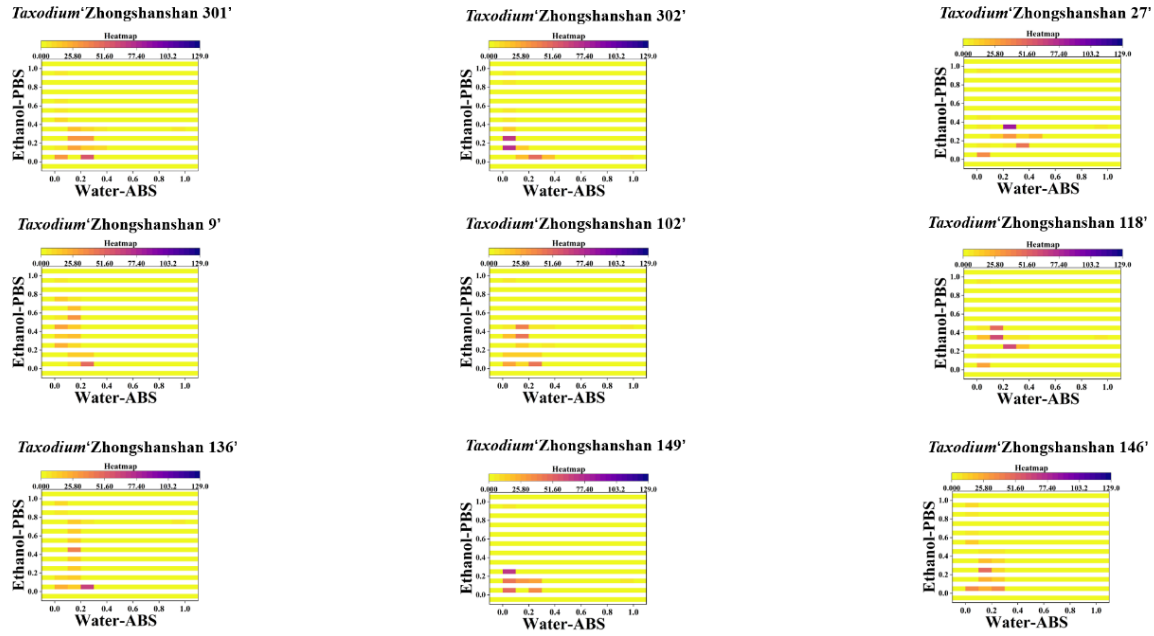

Figure S9. Heatmap of *T*. 'Zhongshanshan 301', *T*. 'Zhongshanshan 302', *T*. 'Zhongshanshan 27', *T*. 'Zhongshanshan 9', *T*. 'Zhongshanshan 102', *T*. 'Zhongshanshan 118', *T*. 'Zhongshanshan 136', *T*. 'Zhongshanshan 125' and *T*. 'Zhongshanshan 146' combining the signals collected under ABS for the water extracts and under PBS for the ethanol extracts.
